# Advancements in ToF-SIMS imaging for life sciences

**DOI:** 10.3389/fchem.2023.1237408

**Published:** 2023-08-24

**Authors:** Feifei Jia, Xia Zhao, Yao Zhao

**Affiliations:** ^1^ National Institutes for Food and Drug Control, Beijing, China; ^2^ Beijing National Laboratory for Molecular Sciences, CAS Research/Education Center for Excellence in Molecular Sciences, CAS Key Laboratory of Analytical Chemistry for Living Biosystems, Institute of Chemistry, Chinese Academy of Sciences, Beijing, China

**Keywords:** ToF-SIMS, life science, metabolomics, lipidomics, single cell imaging

## Abstract

In the last 2 decades, Time-of-Flight Secondary Ion Mass Spectrometry (ToF-SIMS) has gained significant prominence as a powerful imaging technique in the field of life sciences. This comprehensive review provides an in-depth overview of recent advancements in ToF-SIMS instrument technology and its applications in metabolomics, lipidomics, and single-cell analysis. We highlight the use of ToF-SIMS imaging for studying lipid distribution, composition, and interactions in cells and tissues, and discuss its application in metabolomics, including the analysis of metabolic pathways. Furthermore, we review recent progress in single-cell analysis using ToF-SIMS, focusing on sample preparation techniques, *in situ* investigation for subcellular distribution of drugs, and interactions between drug molecules and biological targets. The high spatial resolution and potential for multimodal analysis of ToF-SIMS make it a promising tool for unraveling the complex molecular landscape of biological systems. We also discuss future prospects and potential advancements of ToF-SIMS in the research of life sciences, with the expectation of a significant impact in the field.

## 1 Introduction

Secondary Ion Mass Spectrometry (SIMS) is a powerful analytical technique for surface analysis and characterization of materials. The mature instrument was first introduced in the 1960s and has since become a valuable tool in materials science, planetary geology, surface chemistry, and biological sciences (Williams, 1985). SIMS involves the bombardment of a sample with a high-energy primary ion beam, which interact with a few layers of atoms at the surface and therefore caused the emission of ions, termed secondary ions(SI) for distinguishing from the primary ions, and neutral particles from the surface of the sample. Analyzed by a mass spectrometer, the SI may provide valuable insights into the composition and structural characteristics of the sample’s surface components. SIMS offers several advantages over other surface analysis techniques. It provides high spatial resolution (tens of nanometer level) and can analyze very small surface areas, down to the micrometer scale (Kim et al., 2015; Wirtz et al., 2015). It can detect a wide range of elements, including both metals and non-metals, and can provide information about the chemical bonding and molecular information of the sample surface (Jia et al., 2021; Priebe et al., 2021; Priebe and Michler, 2023; Zimmermann et al., 2023).

There are two SIMS techniques: dynamic SIMS (represented by NanoSIMS) and static SIMS (represented by ToF-SIMS). Although both methods involve analyzing SI emitted from the sample’s surface, they differ in their primary ion beam operation mode and the mass analyzer (Kraft and Klitzing, 2014; Giardina et al., 2018). ToF-SIMS uses a pulsed primary ion beam within the energy range of a few keV to tens of keV, while NanoSIMS uses a continuous primary ion beam of a few tens of keV. The mass analyzer needs to adapt to the primary ion beam being pulsed or continuous, which is a pulsed ion beam for ToF-SIMS, and continuous beam for the magnetic sector of NanoSIMS (McDonnell and Heeren, 2007). Moreover, ToF-SIMS often uses single-atom and clustered primary ions and nanoSIMS usually uses only single atom primary ions. Clustered primary ions is in favor of generating molecular ions or large fragments, while high energy single atom primary ions tend to generate monoatomic and diatomic secondary ions (for example, C, N, Ca, OH, CN, C2, NO). Continued primary ions usually lead to higher sputter rate and higher sensitivity than pulsed ones. Therefore, ToF-SIMS provide information about both the elemental composition and molecular ions or fragments of the samples, while NanoSIMS is usually used for elemental and isotopic composition analysis (Table 1).

**TABLE 1:** Differences between ToF-SIMS and NanoSIMS.

	ToF-SIMS	NanoSIMS
Energy of Primary Ion Source	A few keV to Tens of keV	Tens of keV to Hundreds of keV
Mode of Primary Ion Source	Pulsed	Continuous
Mass Analyzer	Time of Flight	Magnetic Sector
Types of Secondary Ions	Mainly Molecular Fragments and Some Molecular Ions	Mainly Elemental/Isotopic Composition and Some Molecular Fragments
Spatial Resolution	Tens to Hundreds of Nanometers	Tens of Nanometers
Sensitivity	High (ppm to ppb)	Higher (ppb)

**FIGURE 1:**
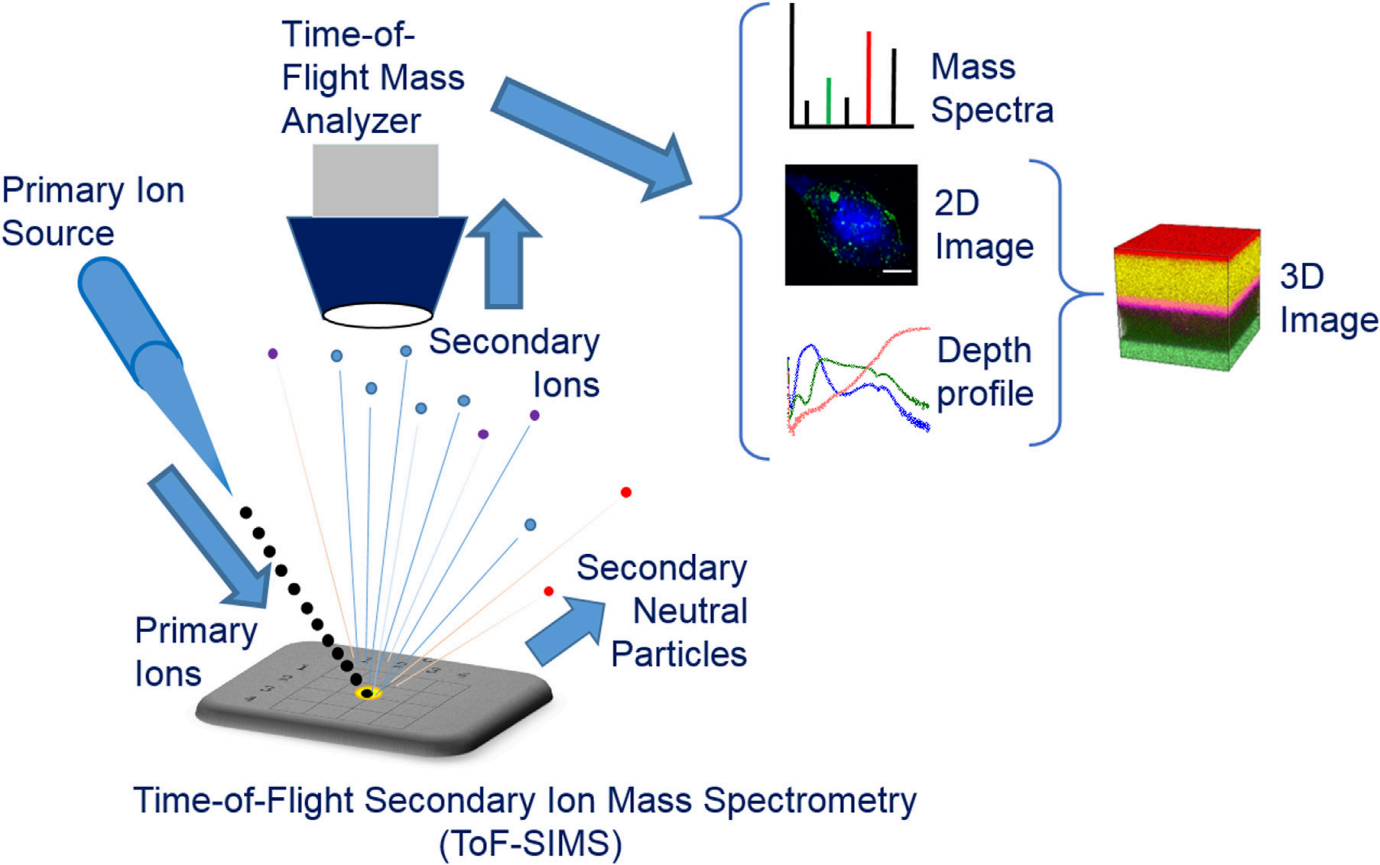
A schematic diagram illustrating the fundamental principles of ToF-SIMS.

ToF-SIMS (Figure 1) is primarily used for the analysis of a sample’s elemental and molecular composition and special distribution, by providing mass spectra, 2D surface images, and depth profiling. Mass spectrometry is the fundamental function of ToF-SIMS, where a focused primary ion beam bombards on the sample surface following a 2D matrix, generating SI that are extracted for *m/z* analysis in mass analyzer. The mass spectrum for each pixel can be obtained and accumulated by multiple scans. The integrated mass spectra provide information about the elements and molecules on the examined area of the sample surface (several atomic layers). Thereafter, by selecting one or more interesting peak (*m/z* signal) from the mass spectra, two-dimensional images can be obtained by data extraction and reconstruction. Then any signal of interest can be extracted and its intensity and loci can be integrated, resulting in a two-dimensional image that provides information on element/molecule distribution with intensity on the sample surface. The lateral resolution of two-dimensional imaging can reach 50–60 nm (Sakamoto et al., 2008; Priebe and Michler, 2023), and the image acquisition can reach a maximum pixel frequency of 50 Hz, with imaging areas ranging from μm^2^ to mm^2^. Three-dimensional analysis applies dual-beam ion sources for both analysis and depth profiling, where a sputter ion beam is used to, etch the sample surface, forming a small crater, while a pulsed ion beam is used to analyze the center of the sputtered crater to give a series of 2D information as mentioned above. Through depth profiling, researchers can also obtain the ion intensity of the sample as a function of depth, with a resolution of less than 1 nm at best with organic multilayers. By combining the data from depth dependent mass spectrometry and 2D images, three-dimensional imaging of sample composition can be achieved, providing information on sample morphology, structure, defects, and others.

Based on its versatile capabilities, ToF-SIMS has found wide-ranging applications in diverse fields, including materials science, surface chemistry, biotechnology, and nanotechnology (Cersoy et al., 2012; Seah et al., 2014; Jia et al., 2019; Guo et al., 2020; Zhang et al., 2021; Cheng et al., 2023). In recent years, it has emerged as a valuable tool for studying biological systems, encompassing the imaging of biological cells and tissues, as well as the analysis of proteins and lipids. The recent advancements in ion sources, sample preparation techniques, and data analysis programs have further expanded the applications of ToF-SIMS.

This review offers a basic overview of ToF-SIMS, and an update of the latest technological advancements of the instruments that benefit the analysis of biological samples. Meanwhile, the relevant applications of ToF-SIMS in the field of life science are introduced, especially in the areas of metabolomics, lipidomics, and single cell imaging, showing that ToF-SIMS has a broad application prospect in the research field of life science.

## 2 Development and status of bioimaging technology in ToF-SIMS

ToF-SIMS has garnered considerable attention in the field of bioimaging owing to its extraordinary high spatial resolution, sensitivity and specificity in detecting molecular species on the surfaces of biological samples. Since the late 1990s, ToF-SIMS has been employed for imaging diverse biological samples (Colliver et al., 1997), including cells, tissues, and biomaterials (Siekkeri Vandikas et al., 2019; Noun et al., 2022). The ability of ToF-SIMS to provide high spatial resolution and molecular information has endowed it a valuable tool for unraveling the molecular mechanisms underlying various biological processes. Over the years, the ToF-SIMS instruments and data analysis methods have undergone significant advancements, leading to improved capabilities for studying biological samples at the molecular level. Therefore, ToF-SIMS has become a potent technique with diverse applications in the field of biological applications.

### 2.1 Primary ion beam

The primary ion beam used in ToF-SIMS is a high-energy beam of ions that strikes the sample surface, causing the ejection of SI that are subsequently detected and analyzed with a mass spectrometer (Agüi-Gonzalez et al., 2019). The choice of primary ion beam is critical as it determines the type and quality of information that can be obtained from the sample (Massonnet and Heeren, 2019).

**FIGURE 2:**
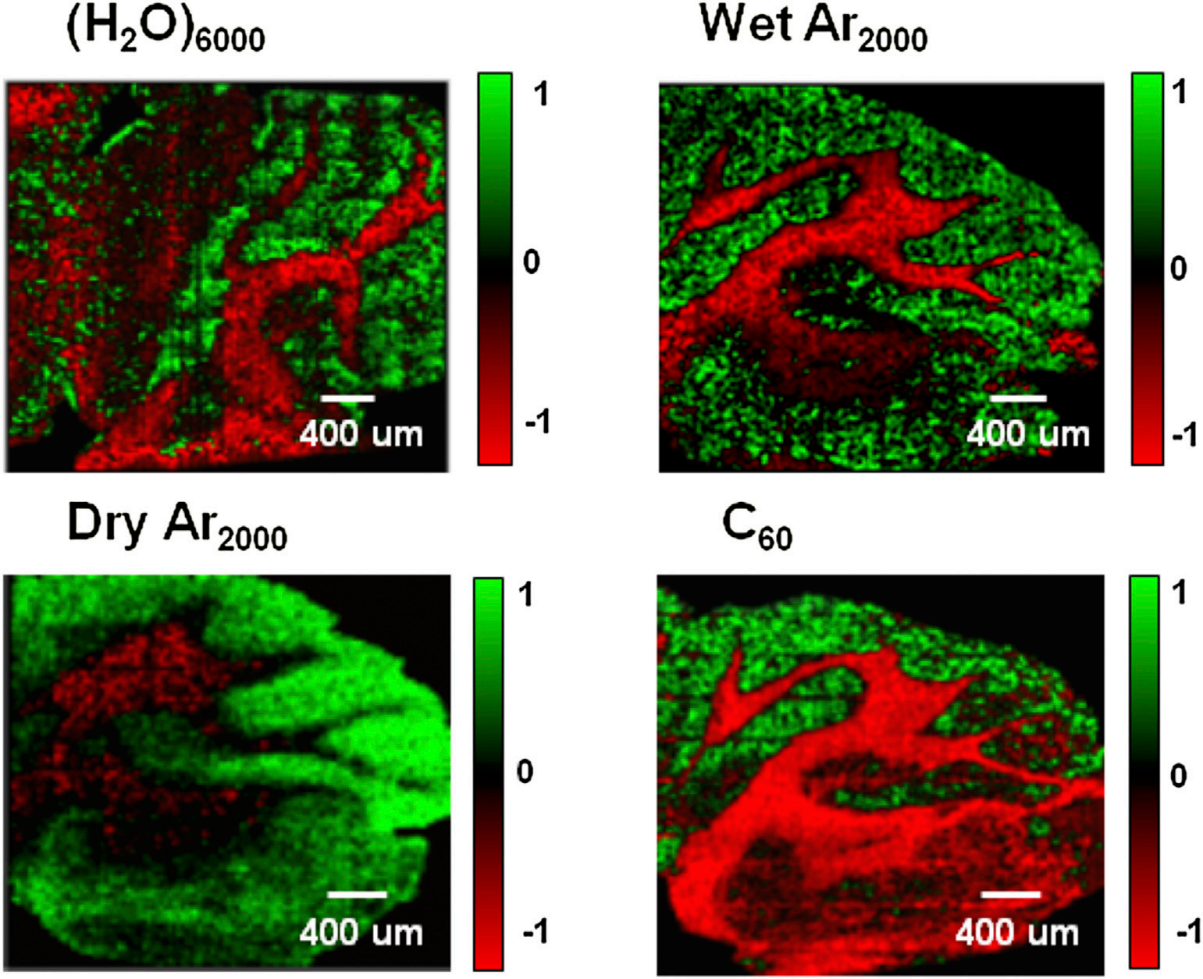
Imaging principal component analysis (PCA) images of the mouse brain cerebellum using four different cluster ion beams: 20 keV (H_2_O)_6000_^+^, 20keV (H_2_O)Ar_2000_^+^, 20 keV Ar_2000_^+^ and 20 keV C_60_^+^. These images show the clear separation between the grey and white matter of the brain. PCA analysis using MATLAB yielded eight principal components (PCs) that capture most of the spectral variance within the dataset. The PCA results are visualized as color density plots representing the scores from each PC. Green pixels indicate positive loadings, while red pixels represent negative loadings. The PC loadings plots confirm that one of the main contributors to the variance between white and grey matter is the cholesterol [M+H−H_2_O]^+^ ion at *m/z* 369. The area covered by each image is 4 mm × 4 mm with a total ion dose of 1 × 10^12^ ions cm^−2^. (Berrueta Razo et al., 2015).

Early ToF-SIMS systems used mono-atomic metal ion beams, such as Ga^+^ or In^+^ ions, which generated high-intensity beams. However, these beams severely damage organic and biological molecules, resulting in poor efficiency in producing secondary molecular ions from biological samples at high *m/z* ranges (Todd et al., 2001; Roddy et al., 2002a; Li and Hirokawa, 2003). To address this limitation, researchers have developed alternative primary ion sources, including cluster ion sources (Mas et al., 2008; Winograd, 2018). Cluster ion sources generate primary ions with lower kinetic energy per atom, so as to cause less damage to biological molecules. Lower energy per atom can only break the intermolecular interaction, which therefore increases the secondary ion yield of organic/biological molecules. Other merits of cluster ion sources include more uniform energy distribution and weaker intermolecular interactions, which also facilitate the analysis of biological samples (Heeren et al., 2006; Vickerman and Winograd, 2015). Cluster ion beams, such as Au_n_^+^, Bi_3_^+^, C_60_^+^, (H_2_O)_n_^+^, (CO_2_)_n_^+^, and Ar_n_^+^, have been widely used in ToF-SIMS bioimaging applications (Berrueta Razo et al., 2015). Four kinds of cluster ion beams used as primary ion sources to image the mouse brain tissue, the principal component analysis (PCA) score image is shown in Figure 2, and these images show the clear separation between the grey and white matter of the brain. A comparison of these cluster primary ion sources is shown in Table 2. The use of these cluster ion beams in ToF-SIMS bioimaging has greatly expanded the scope of biological research and provided a more comprehensive understanding of complex biological questions.

**TABLE 2:** A comparison of cluster primary ion sources for the analysis of biomolecules.

Primary ions	Cluster size	Energy (keV)	Imaging resolution	Selected application area	References
Au_n_^+^	3–400	10	< 100 nm	Biological and polymer material imaging, molecular depth profiling	Touboul et al., 2004; Tempez et al., 2004
Bi_n_^+^	3, 5, 7…	25–35	< 100 nm	Organic and inorganic surface analysis, including 2D and 3D imaging	Touboul et al., 2005
Ar_n_^+^	1,000–6,000	5–40	1–10 μm	Biomolecule imaging; Depth profiling for organic materials	Angerer et al., 2015; Phan et al., 2015; Sheraz née Rabbani et al., 2015
(H_2_O)_n_^+^	1,000–28,000	10–70	1–10 μm	Biomolecule imaging	Sheraz née Rabbani et al., 2015; Dimovska Nilsson et al., 2021; Sheraz et al., 2019
(CO_2_)_n_^+^	3,500–7,000	10–70	8–16 μm	Biomolecule imaging	Dimovska Nilsson et al., 2021; Tian et al., 2017; Sheraz et al., 2019
C_60_^+^	60	10–40	250 nm	Single cell analysis, 3D imaging	Wong et al., 2003

Au_n_^+^ ion beam have been utilized for imaging biological and polymer materials, molecular depth profiling (Davies et al., 2003; Cheng et al., 2007), obtaining quasi-molecular ion peaks or larger fragment peaks of high-molecular-weight biomolecules such as phospholipids, with a spatial resolution of approximately 500 nm. Micron resolved SIMS images of lipids in biological tissue sections were obtained with Au_3_^+^ gold clusters (Touboul et al., 2004). Gold clusters of Au_400_^4+^ has been demonstrated to provide increased molecular ion yield, a higher signal-to-noise ratio of ion peaks, and little damage to surface, as observed in purified biological samples (Tempez et al., 2004). Meanwhile, the implanted Au particles have exhibited an additional capability as a highly sensitive matrix in the analysis of lipids and proteins from brain tissue. Au_400_^4+^ was also used to image the lipid components across a rat brain sagittal section; the results showed the similar pattern and similar relative intensities when compared to matrix assisted laser desorption ionization-mass spectrometry, making it as an attractive probe for sub micrometer matrix-free molecular mapping of native surfaces (Fernandez-Lima et al., 2011).

Similarly, Bi_3_^+^ ion beam, which is the most commonly used liquid metal ion gun (LMIG), offers higher sensitivity than Au_3_^+^ ion beam and is capable of obtaining quasi-molecular ion peaks or larger fragment peaks of biomolecules (Kollmer et al., 2013; Henss et al., 2018; Schneider et al., 2020). Moreover, the spatial resolution has been improved from approximately 500 nm to 200 nm in biological tissues or cells (Touboul et al., 2005). In recent studies, ToF-SIMS with Bi_3_^+^ LMIG coupled with Ar_1500_^+^ as a sputter ion beam was utilized to image rat brain sections, showcasing the potential for enhanced sensitivity in 3D ToF-SIMS biological imaging (Bich et al., 2013). Magdalena Marzec and his colleagues applied Bi_3_^+^ LMIG to investigate changes in fatty acids and amino acids in exosomes and ectosomes derived from human pancreatic β-cells under the influence of hyperglycemia, highlighting the utility of ToF-SIMS as a technique for studying selected amino acids and lipid profiles in various extracellular vesicle subpopulations (Marzec et al., 2022).

The use of C_60_^+^ ion beam has shown the ability to obtain quasi-molecular ion peaks or large fragment ion peaks of biomolecules, with significantly higher analytical sensitivity compared to monoatomic ion beams (Wong et al., 2003; Poleunis et al., 2006). This ion beam can be utilized for both sample etching and sputtering, so as to obtain both 2D and 3D imaging results (Fletcher and Vickerman, 2010). It has a lateral spatial resolution of 2–5 μm and vertical resolution down to the sub-nanometer level (Fletcher et al., 2007; Fletcher et al., 2021). Eric Lanni et al. have developed a hybrid MALDI/C_60_-SIMS Q-ToF mass spectrometer, enabling the imaging of intact biomolecules and their fragments in various biological samples. The team achieved a lateral spatial resolution of 10 μm, with the potential for further enhancement to 1 μm which enabled the visualization of outgrowth and interconnections within neuronal networks (Lanni et al., 2014).

Gas cluster ion beam-SIMS (GCIB-SIMS), benefited from its ability to obtain molecular ions or large fragment ions, has been shown great potential of mapping intact biomolecule in tissue and cell samples, such as lipid and metabolomics (Tian et al., 2017; Pareek et al., 2020). Since the novel water cluster ion source was reported by the Vickerman group in 2013 (Sheraz née Rabbani et al., 2015), it has opened up a valuable new opportunity in the analysis of biological molecules. The (H_2_O)_n_^+^ ion beam, focused to 1 μm, can accelerate proton transfer and significantly improve the analytical sensitivity of positive ions, while obtaining quasi-molecular ion peaks or large fragment ion peaks of biomolecules (Sheraz née Rabbani et al., 2015; Sheraz et al., 2019; Dimovska Nilsson et al., 2021). Compared to C_60_^+^ or argon cluster beams, the water cluster beam has demonstrated a remarkable enhancement in positive molecular ion yield, with a substantial 10–100 fold increase observed across a variety of common analytes, thereby increasing the sensitivity to important lipid molecules. It has been used for high-resolution imaging of HeLa cells and rat brain tissue, achieving spatial resolutions as fine as 1 μm.

The (CO_2_)_n_^+^ cluster ion beam, focused to 1 μm, is mainly utilized for tissue-level metabolic and lipidomics research. It is capable of obtaining quasi-molecular ion peaks or large fragment ion peaks of biomolecules, with lateral spatial resolution of 5–10 μm (Tian et al., 2017). The Ar_n_^+^(n = 60–3,000) ion beam exhibits comparable capabilities to the (CO_2_)_n_^+^ ion beam, offering a lateral spatial resolution ranging from 4 to 15 μm, and is predominantly employed in metabolic and lipidomics research at the tissue level (Rabbani et al., 2011; Thopan et al., 2019). The utilization of Ar_n_^+^ clusters in ToF-SIMS enables efficient generation and detection of SI associated with biomolecules and large molecules. For instance, in a recent study, brain biopsy samples from rats that were injected with microspheres were compared with controls without injection to identify differences in important biomolecules associated with microinfarcts (Nakano et al., 2016). Additionally, argon cluster ion beam can serve as a sputter source in dual-source analysis (Rabbani et al., 2011).

### 2.2 Analyzer

The analyzer in ToF-SIMS instruments plays a crucial role in the mass analysis of SI generated from the sample surface. Early models of ToF-SIMS instruments utilized time-of-flight analyzers with parallel-plate ion mirrors or toroidal ion mirrors, providing moderate mass resolution (>10,000 FWHM) and accuracy (low ppm). The introduction of reflection technology has significantly improved mass resolution (>13,000 FWHM) and enabled better separation of low-mass peaks (Green et al., 2006).

The developments in analyzers, such as the analyzer of TOF-SIMS M6, have shown significant progress in recent years. The M6 TOF Analyzer, in particular, provides higher mass resolution than the TOF-SIMS 5, with up to 26,000 FWHM at *m/z* > 200. This enhanced mass resolution allows for the discrimination of closely spaced mass peaks and more accurate (<10 ppm) identification of molecular species, which is crucial in bioimaging applications where the detection and identification of specific biomolecules are paramount.

**FIGURE 3:**
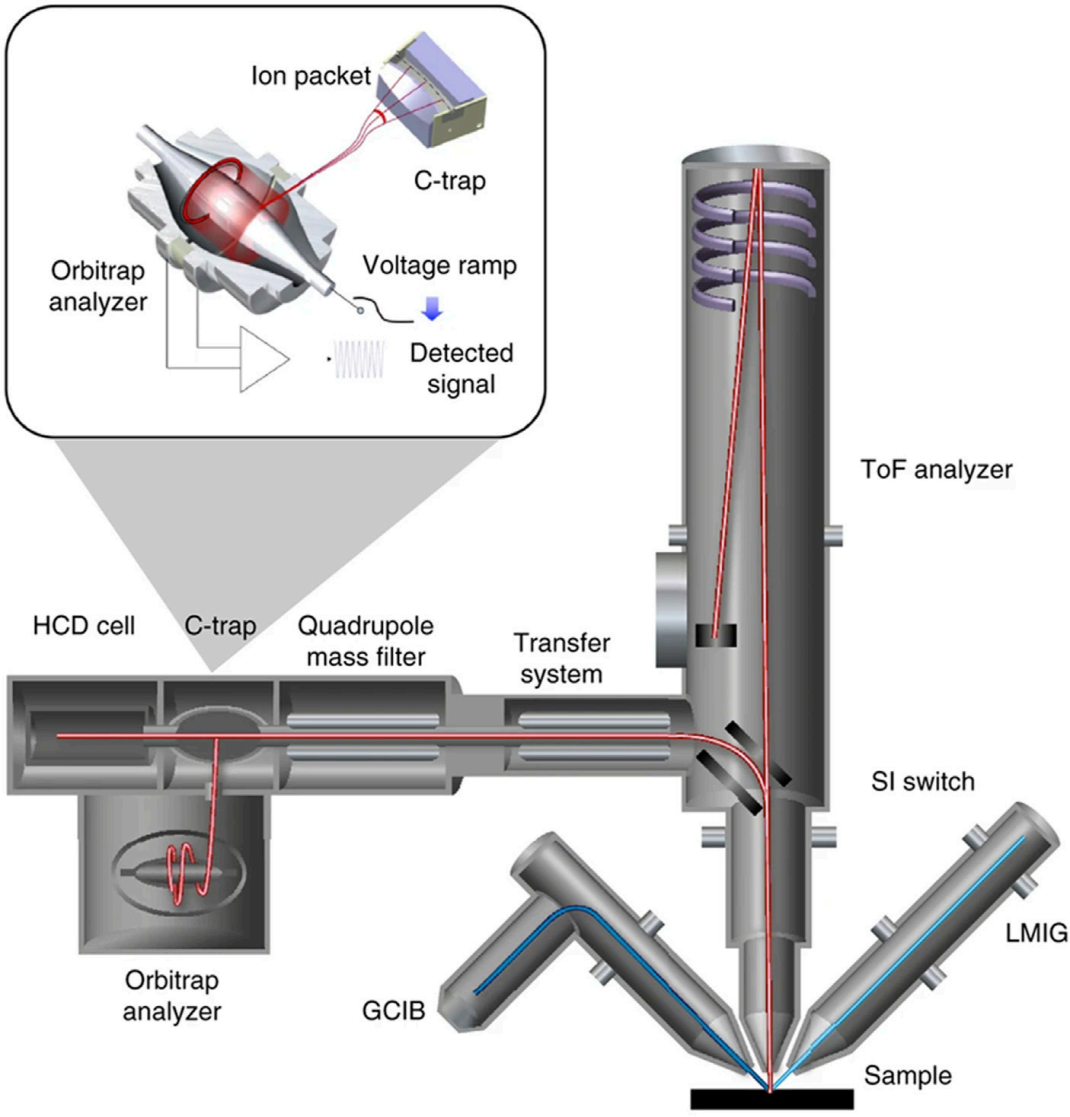
Schematic of the HybridSIMS spectrometer with ToF and Orbitrap mass analyzer (Passarelli et al., 2017).

Hybrid mass analyzers, which combine different mass analyzers, have been increasingly used in ToF-SIMS in recent years. A commonly employed configuration involves the integration of a quadrupole and a ToF mass analyzer (Ens and Standing, 2005; Carado et al., 2008a; Carado et al., 2008b). The quadrupole mass analyzer is used to pre-select ions within a certain mass range before they are injected into the ToF mass analyzer for further analysis. This approach enhances sensitivity and selectivity in ToF-SIMS analysis. Hybrid mass analyzers are particularly useful for analyzing complex samples with a wide range of secondary ion species. By pre-selecting specific mass ranges using the quadrupole mass analyzer, the ToF-SIMS analysis can focus on the species of interest, resulting in improved signal-to-noise ratio and reduced interference from other species. Another type of hybrid mass analyzer combines ToF and Orbitrap mass analyzers (Passarelli et al., 2017) (Figure 3), which provided high speed and high mass resolution (from 15,000 at *m/z* 200 with an acquisition rate >20 spectra up to 480,000 at *m/z* 200 with 1 spectrum) in a broad range of ions, as well as high sensitivity (10^5^ for ratio of total ion counts to noise). The combined capabilities of this hybrid mass analyzer make it a valuable tool for comprehensive and precise analysis of metabolites within biological samples.

## 3 Bioimaging applications in ToF-SIMS

ToF-SIMS is a powerful imaging tool, capable of delivering molecular-level insights into surfaces, thin films, cells, tissues, organs, and whole organisms (Magnusson et al., 2008; Mains et al., 2011; Brison et al., 2013; Yokoyama et al., 2016; Kern et al., 2020; Castellanos et al., 2022; Le et al., 2022). The high sensitivity and spatial resolution of ToF-SIMS make it a valuable tool for the study of biological samples, and the method has been applied to a wide range of samples in the life sciences.

### 3.1 The application of ToF-SIMS in spatial metabolomics

The application of ToF-SIMS in metabolomics has made significant progress in recent years. The ability of ToF-SIMS to detect and image low-mass metabolites with high sensitivity and specificity has made it an increasingly popular technique in the field of metabolomics. By providing high-resolution and label-free imaging of metabolites, ToF-SIMS can reveal complex metabolic processes and interactions that may be difficult to detect using traditional metabolomics techniques, which opened a new research field named spatial metabolomics.

One key advantage of ToF-SIMS in metabolomics is its versatility in detecting various metabolites, such as amino acids, lipids, sugars, and nucleotides (Marzec et al., 2022; Noun et al., 2022; Sjövall et al., 2022). This capability has been demonstrated in several studies, where ToF-SIMS was applied to visualize the metabolites in different cell types and identify potential biomarkers for cell type classification, such as unsaturated diacylglycerol, oleic acid, and linoleic acid, in combination with immunofluorescence (Song et al., 2020). Another study investigated fatty acid profiles in breast muscle tissues of broilers, revealing the impact of fed additives on the composition of fats in the tissues (Marzec et al., 2020).

In addition to spatial imaging, ToF-SIMS can also be used for metabolite identification by comparing spectra obtained from the sample with reference metabolites. This enables researchers to gain insights into the metabolic pathways and biomolecules involved in various biological processes. For example, one study used ToF-SIMS to measure standard metabolites and compare the results with metabolite databases containing ESI-CID MS/MS spectra (Fletcher et al., 2013), assessing the influence of the chemical environment, i.e., the matrix effect, on mass spectra for compound identification and quantification using a mixed metabolite sample.

**FIGURE 4:**
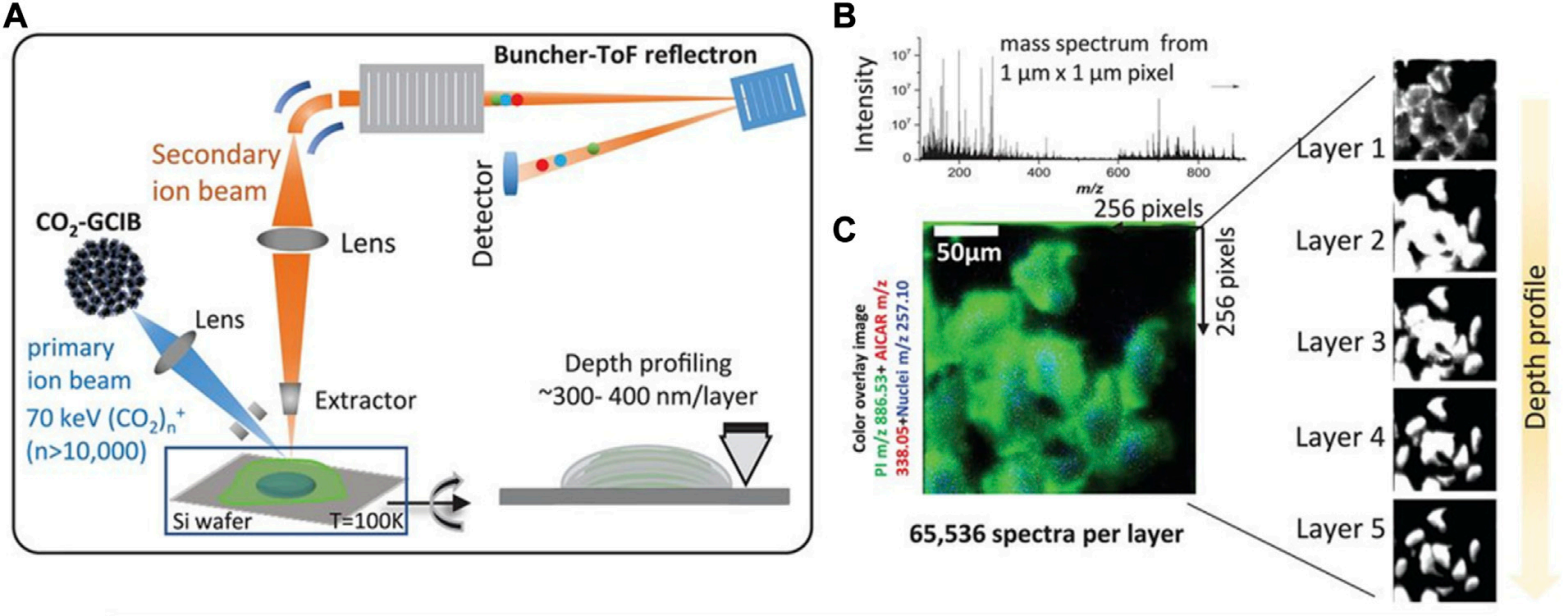
Identification of unique molecular ions of purine nucleotides in the intracellular pool by *in situ* GCIB-SIMS. **(A)** Schematic of GCIB-SIMS imaging of HeLa cells. Imaging uses a finely focused 70 keV (CO_2_)_n_^+^ (n > 10,000) cluster beam to interrogate frozen hydrated HeLa cells three-dimensionally at 1-μm spatial resolution. Coupled with a buncher-ToF and direct-current beam setup, maximum spatial resolution and mass resolution can be retained. A pixel-by-pixel analysis was performed across a lateral field of view of 256 mm × 256 mm. **(B)** Mass spectra in the *m/z* range 0–900 were recorded for each pixel. **(C)** A composite two-dimensional colored image was generated combining the signal across all the layers PI (38:4; green) at *m/z* 886.53, phosphate-sugar backbone at *m/z* 257.10 (blue) from nucleotides, and ^15^N-enriched DNPB intermediate AICAR (red). Combination of mass spectral analysis and the spatial distribution of specific cellular signals demonstrates the reliability of the method for *in situ* biochemical studies (Pareek et al., 2020).

Furthermore, ToF-SIMS has the ability to analyze metabolites within single cells, providing high-resolution imaging of individual cells and revealing cellular biosynthesis, which is essential for life and has far-reaching implications for many areas of science and medicine. For example, a study by Nicholas Winograd group used ToF-SIMS to provides a clear demonstration of the presence and function of metabolons within the cell. Pareek et al. (2020) employed a combination of metabolomics and *in situ* 3D imaging using GCIB-SIMS, directly visualized the *de novo* purine biosynthesis of purinosome by a multienzyme complex (Figure 4). The study revealed the presence of nine enzymes within the purinosome, which function synergistically to regulate the synthesis of purine nucleotides, thereby modulating the pathway flux and impacting the ratio of adenosine monophosphate/guanosine monophosphate. The use of high-resolution GCIB-SIMS demonstrated its utility in unraveling complex metabolic processes and providing insights into cellular dynamics.

The combination of ToF-SIMS and artificial intelligence (AI) has emerged as a promising approach for advancing the understanding of cellular metabolism (Yuan et al., 2021; Gardner et al., 2022). AI algorithms can analyze large and complex datasets generated by ToF-SIMS, identifying patterns and correlations that may be difficult for human analysts to achieve. This can accelerate data analysis and interpretation, may further enable the discovery of novel metabolic pathways and biomarkers. One example of the integration of ToF-SIMS and AI in metabolomics is the use of deep learning algorithms to analyze ToF-SIMS imaging data. Xinrong Zhang’s team has developed a spatial single nuclear metabolomics (SEAM) approach based on ToF-SIMS imaging and computational algorithms (Yuan et al., 2021), enabling tissue zoning and cell typing based on the spatial distribution of metabolites. SEAM allows for the identification and clustering of single nuclei by their *in situ* metabolic fingerprints, enabling the exploration of the spatial metabolic profile and tissue histology at the single-cell level, which may lead to a deeper understanding of tissue metabolic organization.

### 3.2 The application of ToF-SIMS in lipidomics

Lipids play a critical role in various biological processes, including signaling, energy storage, and membrane structure. ToF-SIMS emerged as a powerful technique for identifying and quantifying lipids in biological samples, including brain (Amaya et al., 2007), skin (Kezutyte et al., 2013), liver (Debois et al., 2009), heart (Sämfors et al., 2019) etc., related to various research fields such as tumor microenvironment, pathophysiology of traumatic brain injury, and cellular membrane lipid composition (Bich et al., 2014a; Bich et al., 2014b; Sämfors and Fletcher, 2020; Li et al., 2023; Zhang et al., 2023).

One of the key advantages of ToF-SIMS in lipidomics is its ability to provide detailed chemical information on lipid species with high spatial resolution. By using ToF-SIMS imaging, specific lipid species, such as phospholipids, glycolipids, and sphingolipids, can be identified and localized within cells and tissues. This advanced imaging technique facilitates comprehensive insights into the distribution, composition, and interactions of lipids, and allowing for a better understanding of their functional roles in biological processes (Ostrowski et al., 2004; Kurczy et al., 2010; Lshikawa et al., 2016; Dimovska Nilsson et al., 2020; Blank and Hopf, 2021). For example, By employing a (CO_2_)_6k_^+^ GCIB (Dimovska Nilsson et al., 2020), ToF-SIMS imaging has unveiled the heterogeneity of lipids in basal cell carcinoma tissue from patients, elucidating their spatial distribution. This analysis has demonstrated distinct variations in signal intensities among different microtumors, implying the possibility of chemically grading the aggressiveness of individual tumor islands. Using an In^+^ ion beam, frozen fracture sections of the ciliate *Tetrahymena* were bombarded, and a large number of fusion pores were observed during the fusion process, leading to lipid heterogeneity on the cell membrane. In the area of the fused ciliate cell membrane, the low-curvature phosphatidylcholine decreased, but a large number of high-curvature fusion pores appeared, resulting in lipid heterogeneity in the membrane fusion area (Ostrowski et al., 2004). Further research has shown that the formation of new lipid bilayers (characterized by lipid heterogeneity) during single-cell *Tetrahymena* fusion occurs after the overall structural changes of the cell membrane, or the formation of lipid bilayers is a consequence rather than the cause of membrane structural changes (Kurczy et al., 2010).

**FIGURE 5:**
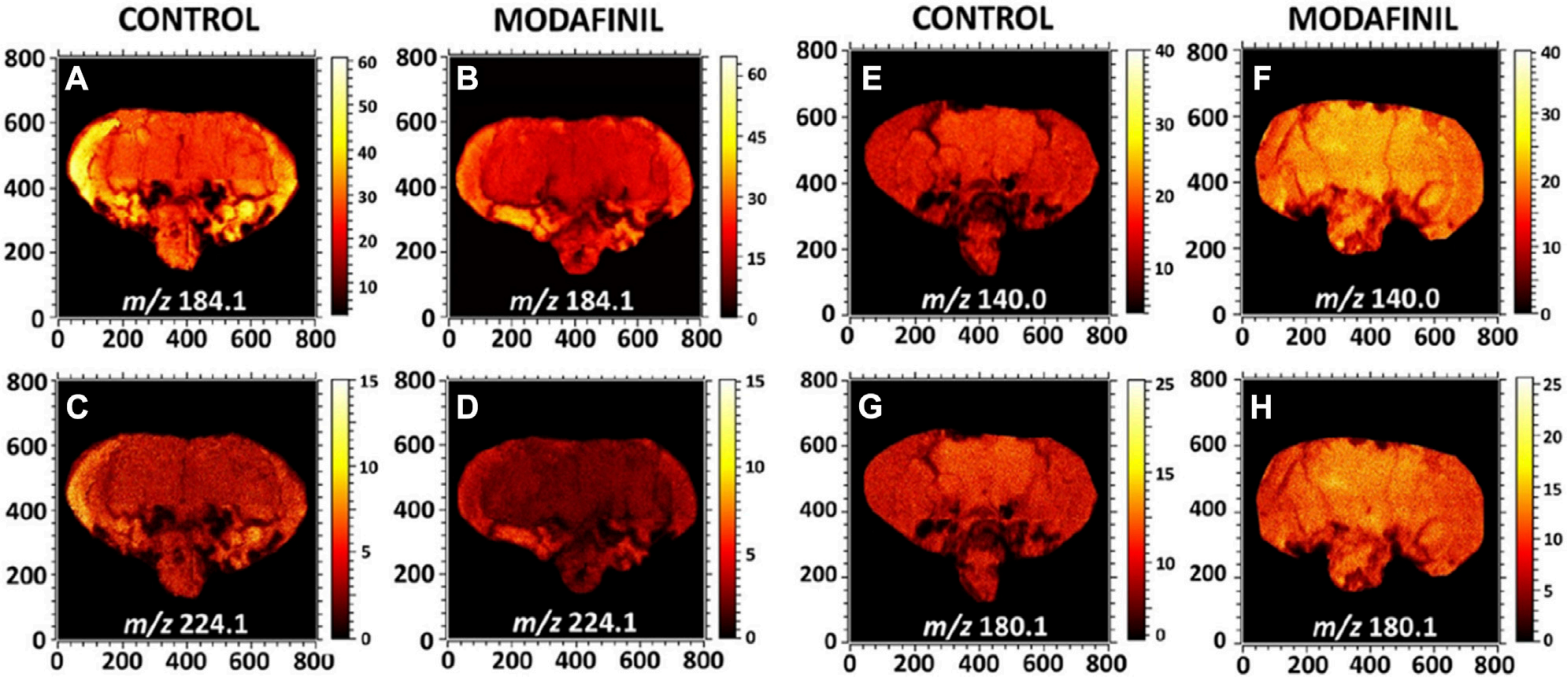
ToF-SIMS images display the distribution of lipid headgroup fragments in fly brain sections of control and modafinil treated flies imaged by ToF-SIMS. **(A,B)** Ion images of the PC and SM head group at *m/z* 184.1 in the positive ion mode; **(C,D)** PC fragment at *m/z* 224.1 in the positive ion mode; and **(E–H)** PE fragments at *m/z* 140.0 and 180.1 in the negative ion mode. All the images were recorded with the ToF-SIMS V instrument equipped with 25 keV Bi_3_^++^ as a primary ion beam. The primary ion beam current was 0.3 pA and the total ion dose was 2 × 10^12^ ions cm^−2^ (Philipsen et al., 2021).

Additionally, ToF-SIMS imaging has been used to investigate lipid changes in response to drug treatments in *drosophila melanogaster* brain tissue, providing insights into drug mechanisms and toxicity (Philipsen et al., 2018). ToF-SIMS imaging studies have revealed that the lipid composition of the plasma membrane of neuron cells undergoes significant changes following treatment with the neurostimulant tetrodotoxin (TTX) and the sedative drug bicuculline (BIC), indicating that lipidome is involved in the regulation of neuronal function (Agüi-Gonzalez et al., 2021). ToF-SIMS imaging also revealed a significant decrease in the abundance of phosphatidylcholine and sphingolipids in the central brain of fruit flies following modafinil treatment, while the levels of brain phosphatidylethanolamine and phosphatidylinositol increased significantly compared to the control group (shown in Figure 5). This provides a reasonable mechanism underlying the effects of modafinil in the brain and identify potential targets for modafinil (Philipsen et al., 2021). Furthermore, ToF-SIMS imaging has been successful in distinguishing between isobaric lipids, which have the same mass but different chemical structures (Gunnarsson et al., 2010), making it valuable for studying complex lipid mixtures. It is worth noticing that in Figures 2, 5, quantitative comparison between two different mass spectrometry imaging experiments can have problems due to different matrix effects. The color intensities could be misleading and should be verified by spiking isotopically labelled standards to the samples.

Combining ToF-SIMS with complementary imaging methods, like fluorescence microscopy, can provide complementary information and enable comprehensive lipid analysis in biological samples (Van Nuffel et al., 2020; Fletcher et al., 2021; Kaya et al., 2021). A study has shown that variations in the membrane composition may affect the growth, metastasis, and therapeutic effect of tumor cells. ToF-SIMS can detect intact lipid molecules on the cell surface, and the increase of phosphatidylethanolamine species on the cell surface may have an important effect on tumor cells. These findings suggest that increased membrane mobility, enhanced motility, and improved adaptation to the utilization of unsaturated lipids could be important factors contributing to cancer cell proliferation (Fletcher et al., 2021). In addition, by combining ToF-SIMS with matrix-assisted laser desorption ionization (MALDI) imaging mass spectrometry and hybrid SIMS, potential biomarkers have been identified in lung tissue samples from patients with pulmonary arterial hypertension, providing new insights into the pathology of this disease (Van Nuffel et al., 2020). Moreover, the combination of ToF-SIMS with tandem mass spectrometry (MS/MS) allows for more precise lipid identification and imaging, effectively excluding the biomolecular interference (Phan et al., 2017; Castellanos et al., 2019).

### 3.3 The application of ToF-SIMS in single cell imaging

ToF-SIMS exhibits exceptional performance in single-cell imaging owing to its high special resolution, specificity, and sensitivity (Gilmore, 2013; Gilmore et al., 2019). With its capacity to precisely analyze diverse target molecules *in situ*, including elements, lipids, and amino acids, ToF-SIMS enables the study of cellular heterogeneity and function at the single-cell level, and has gained tremendous attention in recent years (Passarelli and Ewing, 2013; Wu et al., 2017; Jia et al., 2021).

**FIGURE 6:**
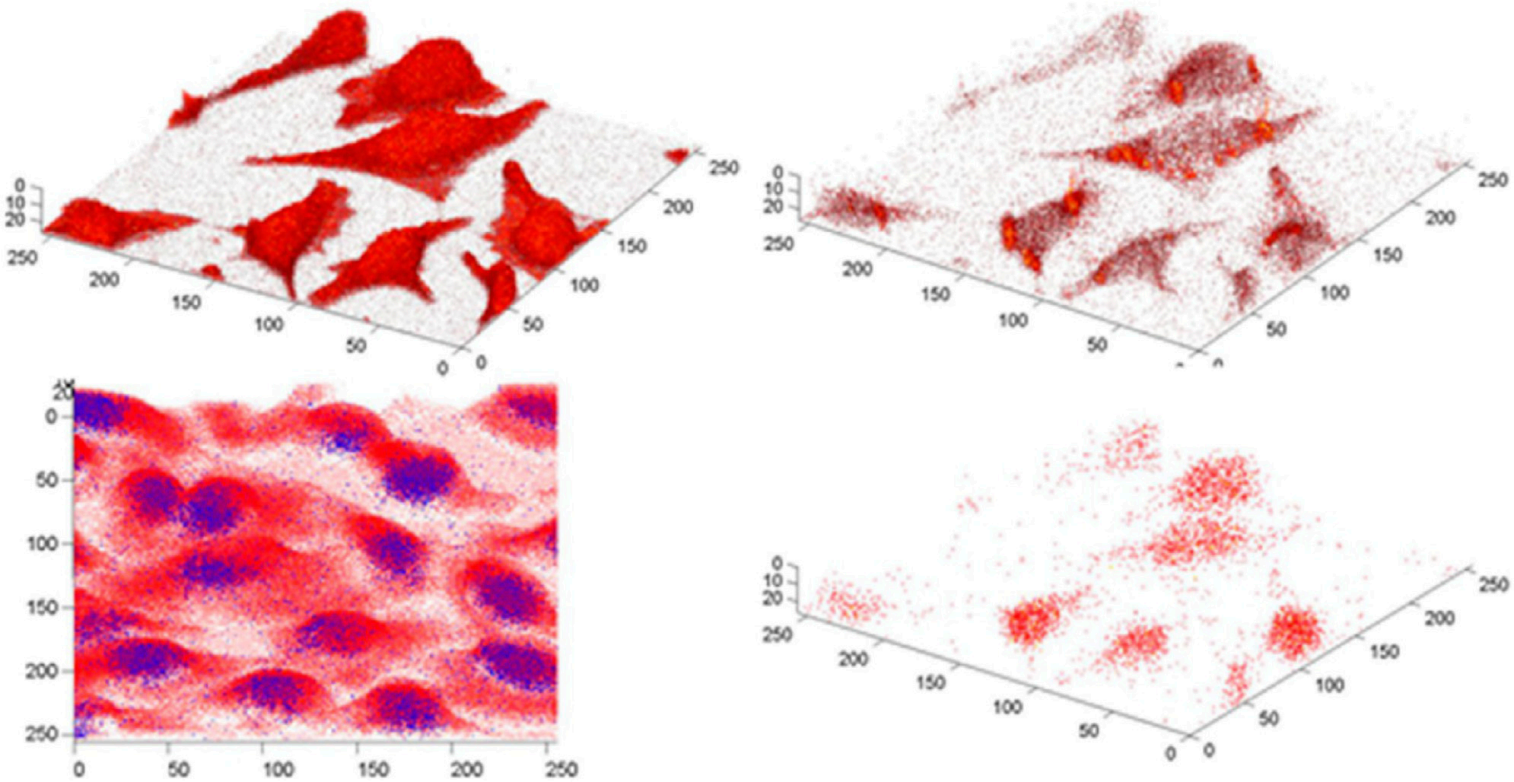
Z-corrected 3D images of BrdU localized within cells using two different LMIG operating modes. The bottom left overlay is obtained from the high mass resolution bunched mode with the BrdU^−^ signal(^81^Br^−^, *m/z* 81) shown in blue and the sum of the C_x_H_y_O_z_^−^ signals(C_2_HO^−^, *m/z* 41, C_2_H_3_O^−^, *m/z* 43, CHO_2_^−^, *m/z* 45, C_3_H_3_O^−^, *m/z* 55, C_2_H_2_O_2_^−^, *m/z* 58, C_2_H_3_O_2_^−^, *m/z* 59, C_3_HO_2_^−^, *m/z* 69, C_3_H_3_O_2_^−^, *m/z* 71) in red. The other three images are burst mode images. The top left: CN^−^(*m/z* 26) + CNO^−^(*m/z* 42), shows the location and shape of each cell; top right: ΣC_x_H_y_O_z_^−^; bottom right: ΣBrdU^−^(^81^Br^−^, *m/z* 81, C_4_H_2_N_2_O_2_^79^Br^−^, *m/z* 189, C_4_H_2_N_2_O_2_^81^Br^−^, *m/z* 191). The bottom left image is 202 × 202 μm^2^ and contains 24 slices. The burst mode images are 165 × 165 μm^2^ and contain 26 slices (Brison et al., 2013).

Achieving optimal sample preparation for ToF-SIMS imaging of single cells can be demanding due to the complex intracellular components, high water content, and high vacuum environment of the instrument. Early single-cell imaging with ToF-SIMS involved analyzing frozen cell samples (Roddy et al., 2002b). The experimental procedure involved culturing the cells on a silicon wafer, followed by the placement of a shard over the cells, and subsequently the whole assembly was frozen in liquid propane. Before imaging, the shard was removed, exposing a fresh surface of the cell samples, and the imaging was performed on a cold sample analysis stage. This approach allowed for imaging of cells at different depths, but finding slices of cells at different depths and selecting regions of interest could be difficult. An alternative approach to avoid cell rupture during freezing is to use freeze-dried cells. However, the potential delocalization of molecules and possible distortion of samples in the freeze-drying process is a problem that cannot be ignored, meanwhile freeze-dried cells can have an uneven cell surface, which may introduce artifacts in 2D imaging, requiring Z-correction or shift correction in 3D imaging (Robinson et al., 2012; Graham and Gamble, 2018). D.S. Castner and his group have developed Z-correction procedures to address this issue, and studied the distribution of the fluorescent dye BrdU in HeLa tumor cells (Brison et al., 2013), as shown in Figure 6. Using a similar method, Fuyi Wang’s team studied the distribution of the newly developed ruthenium-based antitumor drug in MCF-7 cells (Liu et al., 2017). It is worth noting that using a Bi_3_^+^ ion beam for analytical scans for 1,000 frames, exhibited negligible effects on the morphology of freeze-dried cells (Lin et al., 2021).

Other novel cell sample pretreatment methods are also being developed and used to improve the quality of ToF-SIMS imaging. For example, using ionic liquids to preprocess cell samples effectively prevents breakage, deformation, or charging of the samples, and the residual concentration of ionic liquids on the surface is smaller than the detection limit of ToF-SIMS (Yamashita et al., 2022). Another method for 3D reconstruction of biological samples is on-microtomy, which involves combining successive section images and is commonly used with tissue samples but rarely reported in single cells. Alexander Gulin et al. applied this method to fully grown mouse germinal vesicle (GV) oocyte to obtain molecular-specific 3D maps without the impact of surface morphology and uneven etching depth (Gulin et al., 2020). Other sample preparation methods, such as plasma treatment of the sample (Lim et al., 2019), graphene coating of the sample surface (Li et al., 2019), or preparation of the sample using a substrate similar to that in MALDI (Matrix-Assisted Laser Desorption/Ionization) (Körsgen et al., 2016) can effectively remove organic residues from any solution used in the sample preparation process to improve the multiplex molecular ion image of the cell membrane, or to enhance the signal strength of target molecules.

The analysis of metabolomics and lipidomics at the single-cell level presents unique challenges, primarily attributed to the limited quantity of substances within individual cells. As a result, highly sensitive and label-free methods are needed, making ToF-SIMS one of the most effective tools for life science research at the single-cell level (Fessenden, 2016) and has received rising attention (Passarelli and Winograd, 2011; Doerr, 2018; Zhang and Vertes, 2018; Zhang et al., 2022; Barut et al., 2023). Another important application of single-cell imaging is the study of the subcellular distribution of drugs and interactions between drug molecules and biological targets (Passarelli et al., 2015; Hagvall et al., 2021; Akbari et al., 2023). ToF-SIMS has become an important tool for investigating the chemical constituents that contribute to understanding the antibiotic resistance within biofilms. For instance, Anoosheh Akbari et al. utilized sub-micrometer resolution cryo-ToF-SIMS 3D imaging to examine the penetration of the antibiotic ciprofloxacin in *Bacillus subtilis* biofilms (Akbari et al., 2023). Passarelli et al. investigated the distribution of the antiarrhythmic agent amiodarone and its metabolite desethylamiodarone in macrophages (cell line: NR8383) using 3D-SIMS and correlated it with the chemical information of endogenous biomolecules. They found that amiodarone and desethylamiodarone were predominantly present in the cell membranes and subsurface regions, while being absent in the nucleus. These findings shed light on the mechanisms of cellular uptake and distribution of pharmaceutical compounds and their metabolites, providing valuable insights to the mechanisms of action at the single-cell level (Passarelli et al., 2015).

**FIGURE 7:**
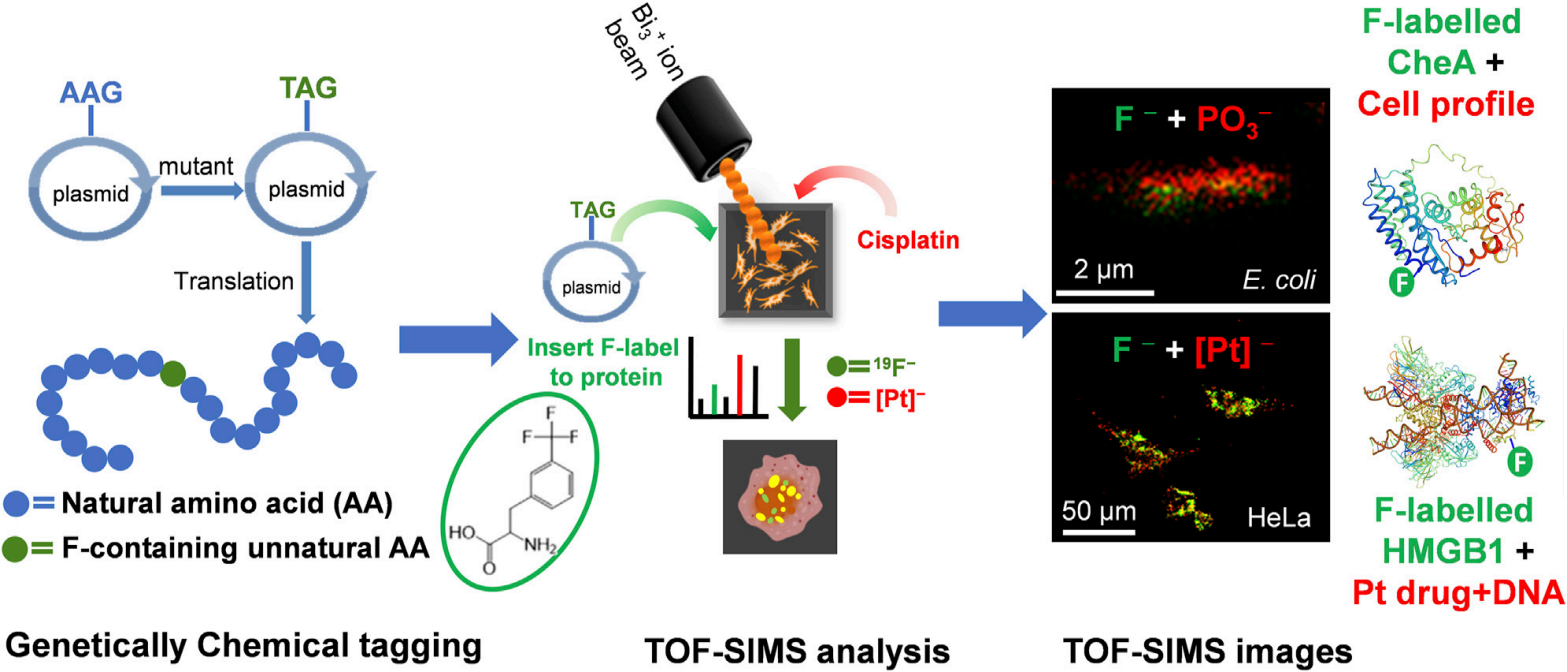
Schematic diagram of visualizing proteins in single cells by ToF-SIMS coupled to genetically encoded chemical tags (Jia et al., 2020). The upper image was generated from the signal of F^−^ (represent protein, green) at *m/z* 19, and signal of PO_3_^−^ (represent the cell, red) at *m/z* 79, while in the bottom figures, the signal of [Pt(CN)]^−^(red) at *m/z* 221 represents cisplatin.

In recent years, Fuyi Wang and his colleagues have systematically investigated the subcellular distribution of several dual-target antitumor compounds based on ruthenium and platinum using ToF-SIMS imaging (Du et al., 2016; Liu et al., 2017; Zhang et al., 2018; Li et al., 2020). They treated A549 cells with ruthenium-containing or platinum-containing antitumor compounds and imaged them using a dual-beam ion analysis. The results revealed that these compounds localized in membranes and nuclei, with their distribution varying significantly with different ligands. These imaging results not only confirm the dual-targeted antitumor activity of these compounds, but also explain the mechanism of their differential antitumor activity. Furthermore, they developed a novel method to investigate the interaction between cisplatin, protein, and DNA *in situ*. They first of all fusion a fluorescent protein with the target protein. Thereafter, by combining confocal fluorescence images and ToF-SIMS images using a custom-designed addressable silicon wafer, they observed, for the first time, the possible formation of the HMGB1-Pt-DNA ternary complex at the single-cell level. This novel approach allowed them to identify that DNA damage induced by cisplatin hindered the interaction between the transcription factor Smad3 and DNA (Lin et al., 2021). Proteins can also be coupled with genetically encoded chemical tags (a fluorine-containing unnatural amino acids) into target protein for direct imaging in single cells by ToF-SIMS (as shown in Figure 7). This approach enables the investigation of protein-drug interactions and has potential applications in drug development (Jia et al., 2020).

Furthermore, nanoparticles as drug carriers have been the focus of research in recent years, and understanding the interaction between nanoparticles and substances in the biological environment is crucial for understanding drug transport mechanisms and evaluating the effectiveness of the treatment. ToF-SIMS technology allows visualization and quantitative analysis of unlabeled drug molecules and nanoparticles in cells (Singh et al., 2020; Wang et al., 2020; Courrèges et al., 2021; Robinson et al., 2021). For example, ToF-SIMS imaging was applied to analyze the targeting ability towards MCF7 breast cancer cells and characteristics of the hyaluronic acid integrated Fe_3_O_4_ nanoparticles (Wang et al., 2020). Biomolecular imaging of cell-nanoparticle interactions is one of important research topics in nanotoxicology. Ajay Vikram Singh et al. proposed 3D distributed nanomaterials in biomolecular aggregation using ToF-SIMS imaging. This novel approach was used to simulate resistance to gold (Au) ionic toxicity in human alveolar A549 cells through intracellular and extracellular biomineralization (Singh et al., 2020).

### 3.4 The application of ToF-SIMS in other fields of life science

ToF-SIMS imaging has demonstrated great potential in various areas beyond metabolomics and lipidomics. For instance, it has been used in fingerprint analysis for drug detection, which has significant implications in forensic and workplace drug testing (Szynkowska-Jóźwik et al., 2021). Water cluster SIMS can detect excreted drug metabolites in fingerprints, allowing for differentiation between drug administration and contamination through contact (Costa et al., 2021).

The implantation of biomaterials *in vivo* is also a research focus of ToF-SIMS (Eriksson et al., 2008; De Carvalho et al., 2020; Kruppke et al., 2020; Malmberg et al., 2021). Implant healing into bone tissue, which involves the growth and fusion of mature bone with the implant, has also been studied using ToF-SIMS imaging. Titanium discs were implanted in rat tibia, and the mineralization process around the implant was examined using ToF-SIMS imaging (Eriksson et al., 2008). For the implants, the *in vitro* effects at the cellular-level and their behavior *in vivo* in animal experiments attracted extensive investigation. The inherent properties of the material, such as the release of biologically active ions, solubility, degradability, and mechanical strength, have a direct influence on cellular responses *in vitro* and bone regeneration process *in vivo*. ToF-SIMS imaging has been able to observe the material degradation and bone regeneration after treatment with gelatin-modified calcium/strontium hydrogen phosphates, providing valuable insights into this process (Kruppke et al., 2020).

ToF-SIMS imaging has found application in the investigation of traditional Chinese medicines (TCMs) and their active ingredients. In order to get high-quality Cordyceps sinensis materials frozen slices, a double-layer embedded sample preparation technique was developed (Liu et al., 2022). ToF-SIMS was then used for analysis and imaging of natural Cordyceps sinensis (NCS) and artificial Cordyceps sinensis (CCS). More than 200 components, including fatty acids and others, were preliminarily assigned and analyzed in NCS and CCS, revealing their high chemical similarity between them. Another application is the non-destructive and rapid detection of TCMs, enabling the evaluation of decoction pieces’ quality by the analysis of ingredients directly in the herbal tissue. For example, the spatial distribution of berberine, epiberberine, coptisine, etc., can be obtained by imaging the cross-section of Coptis rhizome (He et al., 2022). These applications demonstrate the promising analytical potential of ToF-SIMS in TCM research.

## 4 Concluding remarks and future outlook

Despite the significant advancements in the technique and application of ToF-SIMS imaging in life sciences, challenges still arise in various aspects, such as sample preparation, data acquisition and interpretation. One common challenge in ToF-SIMS imaging is sample preparation. The successful analysis of biological samples often relies on optimizing sample preparation techniques to ensure proper preservation, fixation, and embedding of delicate biological structures. Variations in sample preparation protocols can introduce artifacts or affect the integrity of molecular information, leading to potential misleading results. Another challenge lies in the ToF-SIMS technique, where higher special resolution, higher mass resolution, and higher sensitivity are always needed. The development of new ion sources that can form higher level of molecular ions is also in great need. The last but not least, interpretation of complexed data is a great challenge. ToF-SIMS data can provide very detailed molecular information, but severe fragmentation of biomolecules in turn lead to severe difficulty in the interpretation of mass spectra and image datasets. The identification of specific molecular species and their spatial distribution requires the construction of accurate spectral libraries and the development of appropriate data analysis algorithms. Machine leaning based artificial intelligent can play a key role in this field. Hopefully, in the near future, user-friendly software and computational approaches can be developed to handle large datasets, aid data interpretation, and enable more efficient and reliable analysis.

The chemical complexity of biological samples often poses challenges in distinguishing between different molecular species, particularly in complex mixtures, so imaging with high chemical specificity is in urgent need. Additionally, the small size of biological structures, such as organelles and individual cells, requires imaging capabilities with high spatial resolution (Gulin et al., 2020). Therefore, advancements in sample preparation techniques, high sensitivity, high spatial and mass resolution, and high-throughput of ToF-SIMS instruments, as well as integration with other imaging technologies such as fluorescence microscopy and electron microscopy, would provide complementary information and enable more comprehensive analysis of biological samples.

Furthermore, the increasing volume of data acquired from ToF-SIMS imaging requires sophisticated data analysis techniques for accurate interpretation. The integration of emerging data processing methods, such as artificial intelligence, presents an opportunity to develop automated data processing and analysis algorithms capable of handling large datasets and accurately identifying and quantifying specific molecular species. These new challenges lead researchers to a research direction for broader application of ToF-SIMS imaging in the life sciences.

As ToF-SIMS imaging technology continues to advance, it offers the opportunity to investigate biological systems at the molecular level with higher spatial resolution, providing a deeper understanding of different molecular species distribution within subcellular units of cells and tissues, as well as their interactions with biological targets. Additionally, real-time analysis of molecular changes can aid in identifying metabolic pathways and understanding disease mechanisms at the molecular level. The ability to analyze small samples with high sensitivity and resolution makes ToF-SIMS imaging a promising tool for disease diagnosis and surveillance in clinic.

In summary, the ability of ToF-SIMS imaging to analyze biological samples at the subcellular level with high spatial resolution makes it a powerful tool for various applications in the life sciences. With ongoing advancements of technology and the development of new applications, the future of ToF-SIMS in the research of life sciences is promising and holds great potential for advancing biomedical research and clinical applications.
